# Testing a Dutch web-based tailored lifestyle programme among adults: a study protocol

**DOI:** 10.1186/1471-2458-11-108

**Published:** 2011-02-16

**Authors:** Daniela N Schulz, Stef PJ Kremers, Liesbeth ADM van Osch, Francine Schneider, Mathieu JG van Adrichem, Hein de Vries

**Affiliations:** 1 Department of Health Promotion, Faculty of Health, Medicine and Life Sciences, Maastricht University, P.O. Box 616, 6200 MD Maastricht, the Netherlands; 2 School for Public Health and Primary Care (CAPHRI), Maastricht University, Maastricht, the Netherlands; 3 Nutrition and Toxicology Research Institute Maastricht (NUTRIM), Maastricht University, Maastricht, the Netherlands

## Abstract

**Background:**

Smoking, high alcohol consumption, unhealthy eating habits and physical inactivity often lead to (chronic) diseases, such as cardiovascular diseases and cancer. Tailored online interventions have been proven to be effective in changing health behaviours. The aim of this study is to test and compare the effectiveness of two different tailoring strategies for changing lifestyle compared to a control group using a multiple health behaviour web-based approach.

**Methods:**

In our Internet-based tailored programme, the five lifestyle behaviours of smoking, alcohol intake, fruit consumption, vegetable consumption, and physical activity are addressed. This randomized controlled trial, conducted among Dutch adults, includes two experimental groups (i.e., a sequential behaviour tailoring condition and a simultaneous behaviour tailoring condition) and a control group. People in the sequential behaviour tailoring condition obtain feedback on whether their lifestyle behaviours meet the Dutch recommendations. Using a step-by-step approach, they are stimulated to continue with a computer tailored module to change only one unhealthy behaviour first. In the course of the study, they can proceed to change a second behaviour. People in the simultaneous behaviour tailoring condition receive computer tailored feedback about all their unhealthy behaviours during their first visit as a stimulation to change all unhealthy behaviours. The experimental groups can re-visit the website and can then receive ipsative feedback (i.e., current scores are compared to previous scores in order to give feedback about potential changes). The (difference in) effectiveness of the different versions of the programme will be tested and compared to a control group, in which respondents only receive a short health risk appraisal. Programme evaluations will assess satisfaction with and appreciation and personal relevance of the intervention among the respondents. Finally, potential subgroup differences pertaining to gender, age and socioeconomic status regarding the behaviour effects and programme evaluation will be assessed.

**Discussion:**

Research regarding multiple behaviour change is in its infancy. We study how to offer multiple behaviour change interventions optimally. Using these results could strengthen the effectiveness of web-based computer-tailoring lifestyle programmes. This study will yield new results about the need for differential lifestyle approaches using Internet-based expert systems and potential differences in subgroups concerning the effectiveness and appreciation.

**Trial registration:**

Dutch Trial Register NTR2168.

## Background

Cardiovascular diseases (CVD) and cancer are the two major causes of illness and death in the Netherlands as well as in other Western countries. Causal factors of these diseases are partly related to unfavourable lifestyle behaviours, such as smoking, high alcohol consumption, unhealthy eating habits and physical inactivity [1,2]. In the Netherlands, public health guidelines are defined for these health behaviours, i.e., non-smoking, not drinking more than one (women) or two (men) glasses of alcohol a day [3], eating two pieces of fruit per day [4], eating 200 grams of vegetables per day [4], and being moderately physically active for 30 minutes at least five days a week [5]. Approximately three quarters of the Dutch population eats too little fruit and vegetables, nearly half of the population does not meet the recommendation for physical activity, nearly one third smokes and it is estimated that a comparable percentage of people drinks too much alcohol [6,7]. No single behaviour can be ascribed as a sole causal factor in the development of diseases such as CVD or cancer. Recent research shows that about 5% to 10% of the population complies with all these public health guidelines and that 5% to 10% does not comply with any of the five guidelines [8].

Interventions are needed in order to encourage people to adopt a healthier lifestyle. Nowadays, 88.6% of the Dutch population have access to the Internet [9]. This implies that web-based lifestyle interventions have the potential to reach a large number of people [10,11]. Web-based computer tailoring (CT) is a relatively new effective technique for health education. CT is any combination of information or change strategies intended to reach one specific person, based on characteristics that are unique to that person, related to the outcome of interest, and have been derived from an individual assessment [12,13]. Positive aspects of web-based CT programmes are that these can be applied in privacy, for example at home, and at a time the respondent finds convenient [14], and that they can be integrated in other interventions [15]. A CT programme even has some key benefits in comparison to non-tailored materials. It can provide personalized feedback which attracts attention and consists of less unnecessary information than general material [16]. CT messages are usually better read, saved, remembered and discussed with others than non-tailored materials [15,17,18]. CT is cost-effective due to the fact that it can be provided via the Internet and can reach many people [15,19]. CT seems to be more effective in comparison to general messages about health beliefs and health behaviour [15,17,20] as shown by their effects on increasing smoking cessation [21], decreasing alcohol consumption [14,22], increasing fruit and vegetable intake [23-26] and increasing physical exercise [27-30]. In the past, most CT programmes addressed only one behaviour. By now, the focus on multiple behaviours has become increasingly popular [23,31-34]. However, the best strategy for offering health information to motivate the population to change multiple behaviours has not yet been determined.

Two different types of strategies can be distinguished in this respect: a simultaneous behaviour change strategy in which personal advice on multiple lifestyle behaviours is given at the same time; and a sequential behaviour change strategy in which personal advice on multiple lifestyle behaviours is provided consecutively at different points in time. Both strategies have potential advantages as well as disadvantages which may have influence on the effectiveness and appreciation of the tailoring programme. In the simultaneous behaviour change strategy, people receive a lot of information and they may be inclined to choose one behaviour from the range of options and to autonomously change or add to this choice at any time. However, people in the sequential behaviour change condition may perceive a lower level of autonomy since they have to limit themselves to one single behaviour at first [35,36]. Due to the amount of information that is given in the simultaneous behaviour change strategy, participants actually receive more options and choices and this strategy may possibly be more effective for people who are already motivated to change their lifestyle. On the other hand, it is conceivable that individuals in this situation become overwhelmed due to the amount of information, leading to too few energy resources to change [37] and a more negative feeling regarding the programme. This may be particularly true for people who are not ready to make changes in their lifestyle.

Recent research suggests that the probability of enhancing a second behaviour increases when an individual has successfully changed a first behaviour [38]. This means that, for example, an increase in physical activity may lead to improved eating habits [39]. Such synergistic or spill-over effects may occur in both strategies [40], but it is unclear in which strategy such effects are strongest.

The tailoring programme used in the current study is theoretically based on the integrated model for explaining motivational and behavioural change (the I-Change model) [41]. The I-Change model builds on the Attitude-Social influence-Self-efficacy (ASE) model [42], which in turn integrates ideas from social-cognitive models, such as the Theory of Planned Behaviour [43], the Social Cognitive Theory [44], the Health Belief Model [45], and the Transtheoretical Model [46]. According to the I-Change model, the most proximal determinant of behaviour is the intention to perform this certain behaviour. If someone has the intention to perform a specific behaviour, barriers may hinder the actual performance of the behaviour. On the other hand, ability factors (i.e., action plans, goal action) can help to overcome these barriers. Intention is determined by the motivational factors attitude, social influence and self-efficacy, which in turn are influenced by awareness factors (i.e., knowledge, cues to action, risk perception), predisposing factors (i.e., behavioural, psychological, biological, social and cultural factors) and information factors (i.e., message, channel, source).

The main goal of this study is to test and analyse the effects of two different strategies (i.e., sequential and simultaneous behaviour change feedback) using web-based computer tailored technology. The effects will be compared with those of a control group. Furthermore, we will conduct a programme evaluation and explore potential differences in effectiveness and appreciation of the intervention among subgroups (i.e., socioeconomic status- (SES), age- and gender differences) in order to optimize the programme so that it fits better to the individuals' characteristics, needs and preferences.

## Methods

### Design

The current study is related to a larger study, which was approved by the Medical Ethics Committee of Maastricht University and the University Hospital Maastricht (NL27235.068.09/MEC 09-3-016). In that study, we assess level of adoption of the CT programme by focusing on three different exposure rates: *first use *of the intervention, *prolonged use *(staying on the intervention for a substantial period of time) and *sustained use *(revisits to the intervention). Besides this, satisfaction with the service will be carried out.

The present study, which is registered at the Dutch Trial Register (NTR2168), consists of a randomized controlled trial (RCT) including two experimental groups, namely a Sequential Behaviour Tailoring (SeqBT) condition and a Simultaneous Behaviour Tailoring (SimBT) condition, and a control group. The conditions will be described in detail below. A design with a baseline measurement and two follow-up measurements at 12 and 24 months is used.

### Participants, inclusion criteria and procedure

The study is part of an initiative of different Dutch Regional Health Authorities (RHAs) of the provinces North-Brabant and Zeeland. Our CT programme is partly integrated in the *Adult Health Monitor 2009 *which is conducted by the RHAs. With this monitoring instrument, the health status of people living in the Netherlands is assessed. Every four years, a representative sample of the population aged between 18 and 65 years is recruited in order to fill out this questionnaire. It contains the following topics: demographics; aspects of general health (e.g., physical health, mental health, social health, lifestyle behaviours); and health related topics (e.g., social and physical environment) [47]. As part of an additional service, participants completing this monitor online subsequently obtain the opportunity to receive tailored feedback about their health behaviour via the new CT programme. Therefore, some data the person fills out during the *Adult Health Monitor *(e.g., demographics and data regarding the five lifestyle behaviours: smoking, alcohol intake, fruit consumption, vegetable consumption and physical activity) are copied to the CT programme. Before this, participants receive information about the purpose of the current study and an explanation of the use/handling of their data. We ask for written informed consent for their participation in the project. Confidentiality is ensured and it is explained to respondents that they could withdraw participation at any moment.

Approximately three weeks after completing the monitor, participants who are interested in obtaining tailored feedback about their health behaviour receive an e-mail including an invitation to log in to the CT programme by use of their personal login code and password. After successfully logging in, respondents receive the health risk appraisal (i.e., feedback concerning their lifestyle and information about whether they meet the public health guidelines) based on their answers to the *Adult Health Monitor*, and the experimental groups receive some additional questions (i.e., attitude, social influence, preparatory action plans, self-efficacy, coping plans) and personal advice. The different parts of the programme will be described in detail below.

### The web-based CT programme

To deliver personalized advice, three inter-related elements are necessary: a *screening instrument *to measure demographics, health status, health behaviour and behavioural determinants; a *message source file *containing all tailoring messages; and a *computer programme *that offers the possibility to analyse the screening results and to select the correct messages from the message file [15,48].

The intervention programme is developed by means of the "TailorBuilder"-software (OverNite Software Europe B.V., The Netherlands). The TailorBuilder is a web-based instrument that was specifically developed to create and conduct online questionnaires and design and implement tailored advice. Respondents can login via a website on which general information about smoking, alcohol intake, fruit and vegetable consumption, and physical activity is given as well as detailed information about the study, frequently asked questions and a contact form.

The web-based CT programme combines several programmes which have already been proven to be effective in increasing smoking cessation [21], reducing the consumption of alcohol [19], promoting the intake of fruit and vegetables [25], and increasing the level of physical activity [28]. Tailoring algorithms that combine the responses of a person on specific questions with concurrent feedback messages generate highly personalized advice to the respondent which they receive on their screen and that can also be printed. The following two paragraphs describe the questionnaire and the advice components in more detail.

### Measurement instruments

#### Demographic information

The following demographic variables are measured: age, gender, height, weight, marital status, religious background, ethnicity, educational level, current work status, and income.

#### Health status

Respondents are asked if they suffer from high blood pressure and different kinds of diseases, such as diabetes and cancer. To measure quality of life, the SF-12 Health Survey [49,50] and the Mental Health Inventory (MHI-5) [51] are used. To assess symptoms of depression and anxiety, the Kessler Psychological Distress Scale (K10) [52] is used.

#### Lifestyle behaviours

*Smoking *is measured by the abbreviated version of the Fagerström Test for Nicotine Dependence [53]. *Alcohol consumption *is measured by the Dutch Quantity-Frequency-Variability (QFV) questionnaire [54]. *Fruit and vegetable intake *is measured using Food Frequency Questionnaires (FFQ) for fruit and vegetable intake [55]. *Physical activity *is measured by the Short QUestionnaire to ASsess Health-enhancing physical activity (SQUASH) [56].

#### Intention

The intention to change behaviour is measured by one item. This item is an extended version of the 'stages of change' concept [46,57] using an algorithm consisting of ten stages varying from unawareness to maintenance.

#### Attitude

To measure attitude concerning the different health behaviours, participants have to indicate on a five-point scale to what extent they 'totally disagree' or 'totally agree' with a total of six statements. *Pros *(i.e., advantages) of the health behaviour under consideration are assessed by three items, such as "Regular physical activity is good for my health". *Cons *(i.e., disadvantages) are assessed by another three items, such as "Regular physical activity costs a lot of time".

#### Social influence

Social influence is measured by three different concepts, namely social norms, social modelling and social support. *Social norms *are assessed by one statement regarding the opinion of the direct environment (i.e., partner, family members, friends and colleagues) concerning each health behaviour. Respondents are asked to complete statements, such as "According to the people within my direct environment...", by choosing one of five options ranging from "I certainly should not smoke" to "I certainly should smoke". *Social modelling *is assessed by asking the participants how many people of their direct environment engage in the healthy behaviour, e.g., eating at least two pieces of fruit every day. A five-point scale is used, ranging from 'everybody' to 'nobody'. *Social support *is measured by one item assessing the degree of support respondents receive from their direct environment to engage in the healthy behaviour, on a four-point scale, ranging from 'yes, they support/stimulate me a lot' to 'no, they do not support/stimulate me at all'.

#### Preparatory plans

Preparatory plans are measured by three items for each health behaviour, such as "I intend to become a member of a sports club". Respondents have to specify whether they have the intention to use a specific preparatory plan in order to adopt the particular healthy lifestyle. A five-point scale is used, ranging from 'yes, definitely' to 'no, definitely not'.

#### Self-efficacy

Self-efficacy is measured by means of six items. Participants have to indicate on a five-point scale to what extent they feel able/unable to engage in or refrain from the key health behaviour when encountering difficult situations (i.e., situations that might lead to the performance of the unhealthy behaviour), e.g., "I am able to drink not more than two glasses of alcohol when I am at a party".

#### Coping plans

Coping plans are assessed by asking participants to indicate on a five-point scale to what extent they agree/disagree with a total of six statements. Every statement deals with a specific plan regarding how to cope with difficult situations, e.g., "I have made a plan to be sufficiently physically active even when I have a great deal to do". These questions are based on the difficult situations used for the self-efficacy items.

### Feedback procedure

The message source file generates the tailored pieces of advice about the health behaviours, attitude, social influence, preparatory plans, self-efficacy and coping plans. Hence, respondents receive tailored pieces of advice on their computer screen immediately after completion of each part (concept) of the questionnaire in order to avoid a very long text at the end of the programme. Either text-based messages or graphic feedback are given, in addition to pictures. Besides, in some pieces of advice, links to detailed information are added for people who want to receive more information about certain topics. Tailor formulas are used in order to determine which advice the respondent receives. After completion of the programme, respondents receive an overview of all the pieces of advice they have received while doing the test. This total advice can be printed and is also sent to the respondent's e-mail address, so that it can be re-read at any time. The programme consists of two kinds of feedback, namely (1) a health risk appraisal and (2) personal advice on psycho-social constructs.

#### Health risk appraisal

The first part of the feedback consists of a health risk appraisal which comprises information about the Dutch guidelines defined for the health behaviours. Respondents obtain feedback via text that is illustrated for each behaviour with a green, orange or red traffic light to indicate the level of adherence to the Dutch guideline [58]. A green light implies adherence to the guideline; an orange light implies non-adherence by being close to the healthy level whereas a red light represents non-adherence. Respondents receive an explanation regarding their score for each behaviour. For instance, "You drink approximately 5 glasses of alcohol per day. This means that you do not meet the Dutch guidelines defined for acceptable alcohol consumption." Furthermore, respondents can click on links to receive additional and more detailed information about the guidelines and the specific health behaviour. For example, they can receive information about the relation between alcohol intake and pregnancy and the related danger for the unborn child. Furthermore, respondents' scores are depicted graphically via a bar chart that compares the respondents' behaviour (e.g., the number of alcoholic drinks a day) with the guideline for this behaviour. At the end of the health risk appraisal, respondents receive an overview illustrating the lifestyle behaviour status in terms of five traffic lights (i.e., one traffic light per health behaviour) side by side so that they can evaluate their lifestyle at a glance.

#### Personal advice

In the second part of the programme, a progressive scheme of four steps is applied.

##### Step 1

During the first step, emphasis is placed on *attitude*. The advantages and disadvantages of the relevant behaviour are explained, and applied to the beliefs of the respondent. In other words, knowledge is confirmed or corrected, and (new) information is given and deepened.

##### Step 2

During the second step, we focus on discussing *social influences *and explain to the respondent that the social environment may influence the behaviour of the respondent and the other way around. They receive information about how to cope with pressure from others, e.g., how to refuse a cigarette. The importance of social support and of being a good role model is explained. In addition, the possibility of changing the unhealthy behaviour with the aid of others (e.g., increasing the amount of physical activity by taking exercise together with a friend) is also mentioned.

##### Step 3

During the third step, the concept *preparatory action plans *is addressed. Respondents are invited to make action plans in order to prepare their behavioural change. For instance, we recommend that respondents take fruit along when they go somewhere (e.g., to work) to guarantee availability at the moment they want it. When they have indicated their specific plans, they receive positive reinforcement if they have made preparatory plans and additional tips on how to increase the success of their plan. If they indicate that they are not willing to carry out a plan, we try to persuade the person to try out an alternative plan and provide advice and suggestions on how to do so.

##### Step 4

During the fourth step, attention is given to the concepts *self-efficacy *and *coping plans*. A summary is given relating to the situations the respondent indicates that they expect to be difficult and for which difficult situations the respondent has or has not already made a plan. For example, if someone reports drinking alcohol when he or she feels depressed, it is explained that alcohol does not help against negative feelings in the long run and that it would be better to do something other than drinking alcohol in such a situation, for instance, to go out for a walk or to talk about the feelings with a friend. Respondents receive an overview of their perceived difficult situations and their plans in order to facilitate remembering these. If people perceive a situation as difficult, but they have made no plan, we give a number of specific tips on how to behave in a healthy way in this particular situation.

### The two experimental groups and the control group

**Figure 1:**
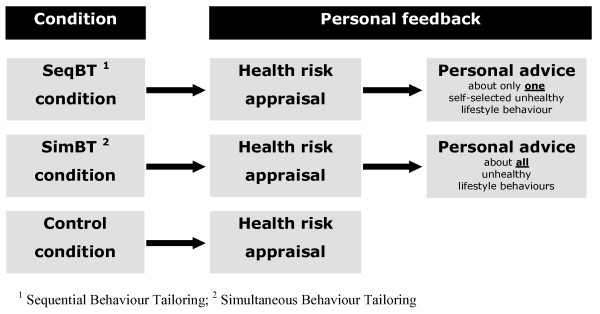
Design at baseline

As presented in Figure 1, the three groups differ in the amount of feedback they receive. The experimental groups have the option to obtain health risk appraisals and personal advice as often as they want whereas the control condition only receives the health risk appraisal once. Every time the experimental groups visit the programme, *ipsative feedback *is given about their health behaviour. This implies that the health behaviour scores of the current visit are compared to the scores of the last visit; feedback is given about a potential change. Furthermore, all scores are saved and illustrated in a graph in order to give the respondent the option to monitor the total change process at a glance. Ipsative feedback is not available for the control group.

#### Sequential Behaviour Tailoring (SeqBT) condition

After receiving the health risk appraisal, individuals in the SeqBT condition are invited to choose one of the health behaviours for which they have received a red or orange traffic light. Respondents are encouraged to change the behaviour for which they are motivated most. After receiving additional questions regarding the various psychosocial constructs of the selected lifestyle behaviour, they receive personal advice on this behaviour which aims at helping to change this health behaviour during the first year of the study.

At the follow-up measurements, individuals in this condition are asked to fill out a questionnaire about all behaviours. Again, they receive the health risk appraisal, including an overview consisting of five traffic lights. In cases where a respondent has successfully changed the selected behaviour, the option to choose a second behaviour in the second year is provided. They are free to select one of the behaviours for which they receive a red or orange traffic light. In cases where respondents have not changed the first behaviour, they will be invited to change the behaviour for which they are motivated most in the second year. This may be the same behaviour as chosen in the first year, but the respondents may also select another lifestyle behaviour for which they have obtained a red or orange traffic light. Afterwards, they receive the questionnaire containing items about perceptions (beliefs) regarding the behaviour they have selected to focus on as well as personal advice.

If respondents fill in the test in the meantime, which means that they re-visit the programme without having received an invitation for a follow-up measurement, they only have to answer the questions concerning the selected behaviour in order to receive ipsative feedback on their chosen topic and personal advice if they are interested.

#### Simultaneous Behaviour Tailoring (SimBT) condition

Upon receiving the health risk appraisal, participants of the SimBT condition are not explicitly asked to select one behaviour which is in contrast to the SeqBT condition. In cases where these participants do not adhere to the recommendation of one or more of the lifestyle behaviours, they obtain additional questions (psychosocial constructs) as well as personal advice on all behaviours for which they receive a red or orange traffic light in the health risk appraisal.

At every follow-up visit/re-visit, individuals are asked to fill out questions about all five lifestyle behaviours. In this way, the programme can check on which behaviours the individual should receive personal advice concerning his or her perceptions. Moreover, they receive ipsative feedback concerning all five behaviours.

#### Control condition

During the first visit of the programme, respondents in the control condition receive the health risk appraisal. Afterwards, this group only receives e-mails requesting them to fill out questionnaires about the five lifestyle behaviours at follow-up at 12 months (T1) and 24 months (T2). The control group does not receive any personal advice throughout the term of this study.

### Primary and secondary outcomes

Our first objective is to compare the effectiveness of the two different strategies for changing lifestyle with each other as well as with a control group.

In addition to the effect studies, we will also gather and analyse data to realize additional outcomes:

#### Process evaluation

Process evaluations will be executed to assess levels of personalization and appreciation and to analyse which elements of the CT programme were most attractive and successful. We aim at gathering process evaluation data to analyse the factors facilitating and hindering the use of the web-based modules. All participants receive questions concerning the *website *(5 items; e.g., "In my opinion, the website is user-friendly"; totally agree - totally disagree), the *health risk appraisal *(12 items; e.g., "The health risk appraisal gives a good overview of my lifestyle"; totally agree - totally disagree) and two *general questions *(i.e., "Do you intend to re-visit the website in the future?"; yes, definitely - no, definitely not; "Do you have any general comments concerning the programme?"; open question). The two experimental groups additionally receive questions regarding the personal advice (15 items; e.g., "The pieces of advice are informative"; totally agree - totally disagree). We will compare the evaluations of the three different groups to analyse differences with regard to the process evaluation data.

#### Subgroup differences

We aim at exploring potential differences between high SES and low SES groups, different age groups, men and women, and healthy and unhealthy people (defined by the items about health status).

### Power analysis

The power analysis is based on detecting 10% differences in behaviour between the different conditions. Power analysis calculations differ per behaviour. Estimations for finding effects on the lifestyle behaviours of smoking and alcohol consumption were the most conservative power analyses resulting in the greatest number of respondents needed. Since approximately 25% to 30% of the population smoke and almost similar percentages are estimated for non-compliance with the alcohol guideline [7], sample size calculations are highly determined by these two groups. In order to be able to find a significant difference of 10% in quit rates and improved behaviour between the conditions, power calculations (alpha=.80; p=.05) indicate that 219 smoking persons per cell are needed, resulting in a total of 657 smokers. Hence, if approximately 25% of the adult population smokes this requires a total study sample of 2,826 persons. A similar conclusion can be drawn for the persons not adhering to the Dutch alcohol recommendations. Correcting for dropout this indicates that the total sample size for this study will be approximately 3,285 persons. The sample will be sufficient to assess changes in the other behaviours (fruit and vegetable consumption and physical activity).

### Attrition prevention

Different strategies are used in order to prevent attrition and to encourage participation in the long run. First, participants are eligible to win prizes. Participants completing the study stand a chance to win one of 300 prizes of 50 Euros. Second, half of the respondents of the experimental groups receive e-mail prompts, including invitations to visit the tailoring programme, every three months, in order to be able to analyse whether pro-active prompting will enhance visiting the website and using computer tailored feedback more intensively. Third, new information is placed on the website monthly in order to keep it up to date and make it attractive for people to re-visit the website.

### Statistical analyses

General descriptive statistics will be used to describe the characteristics of the participants and the main findings concerning the public health guidelines. With regard to the assessment of behavioural effects and differences between the three groups, logistic as well as linear regression analyses will be performed using Statistical Package for the Social Sciences (SPSS) version 15. With regard to the process evaluation, descriptive statistics will be used, such as frequencies, means, standard deviations and ranges. Satisfaction with the programme and ideas for improvement will also be analysed by summarizing the answers to the open-ended question and comparing different attitudes and suggestions. In order to explore potential differences between groups (e.g., low SES versus high SES, age groups, gender), regression techniques and latent cluster analyses will be used.

## Discussion

The results of this study are of importance for the future development and implementation of CT programmes, which aim at stimulating healthy lifestyles in order to prevent chronic diseases. Positively changing a set of health risk behaviours, i.e., smoking, high alcohol consumption, bad nutrition and physical inactivity, is becoming increasingly common since these changes are relevant for the primary prevention of cardiovascular related diseases and cancer [1,2]. Due to the target of several behaviours and health problems, by using a tailored web-based programme, these kinds of programmes are considered to be potentially cost-effective [11].

Internet-based health education programmes have become popular in recent years. To our knowledge, however, few were tested in a RCT to analyse effects on multiple behaviours. Addressing smoking, alcohol consumption, fruit and vegetable intake, and physical activity at the same time, is one fundamental part of recent developments. In addition, we compare a simultaneous behaviour tailoring approach with a sequential behaviour tailoring approach. Hence, we will experimentally test which tailoring lifestyle approach is more effective and appreciated. This study will thus yield results about the need for differential lifestyle approaches. An optimal use of (one of) the different strategies could increase the effectiveness of web-based CT lifestyle programmes. Research is also important in order to assess whether the effectiveness and appreciation of the programme differs between various subgroups.

Furthermore, a unique element of this project is that our CT programme is offered as an additional service of the RHAs in the Dutch regions of North-Brabant and Zeeland within the regular health monitoring they provide to adults. The integration of the CT programme to the *Adult Health Monitor 2009 *brings along various advantages. First, the RHAs may extend and improve their health education task by offering tailored feedback regarding lifestyle behaviours to their respondents. Second, the personal advice may be an extra stimulus for people to participate in the *Adult Health Monitor*. Third, the *Adult Health Monitor *of the two provinces has a total reach of nearly 100,000 inhabitants. Due to the integration of our CT programme into the 'Adult Health Monitor', a large number of people within the Dutch population may be reached.

## Competing interests

The authors declare that they have no competing interests.

## Authors' contributions

HdV, SK and LvO designed and wrote the original proposal. DS, FS and MvA developed the lifestyle programme and execute the studies. DS significantly contributed to writing this paper, while HdV, SK, LvO, FS and MvA were involved in revising the manuscript critically. All authors read and approved the final version of the manuscript.

## Pre-publication history

The pre-publication history for this paper can be accessed here:

http://www.biomedcentral.com/1471-2458/11/108/prepub
